# Impact of caBIG on the European cancer community

**DOI:** 10.3332/ecancer.2011.225

**Published:** 2011-10-03

**Authors:** R Warden

**Affiliations:** 1 European Association for Cancer Research, University of Nottingham, Nottingham, UK

## Abstract

The cancer Biomedical Informatics Grid (caBIG) was launched in 2003 by the US National Cancer Institute with the aim of connecting research teams through the use of shared infrastructure and software to collect, analyse and share data. It was an ambitious project, and the issue it aimed to address was huge and far-reaching. With such developments as the mapping of the human genome and the advancement of new technologies for the analysis of genes and proteins, cancer researchers have never produced so much complex data, nor have they understood so much about cancer on a molecular level. This new ‘molecular understanding’ of cancer, according to the caBIG 2007 ‘Pilot Report’[1], leads to molecular or ‘personalised’ medicine being the way forward in cancer research and treatment, and connects basic research to clinical care in an unprecedented way. But the former ‘silo-like’ nature of research does not lend itself to this brave new world of molecular medicine—individual labs and institutes working in isolation, “in effect, as cottage industries, each collecting and interpreting data using a unique language of their own”[2] will not advance cancer research as it should be advanced. The solution proposed by the NCI in caBIG was to produce an integrated informatics grid (‘caGrid’) to incorporate open source, open access tools to collect, analyse and share data, enabling everyone to use the same methods and language for these tasks.

caBIG is primarily a US-based endeavour, and though the tools are openly available for users worldwide, it is in US NCI-funded cancer centres that they have been actively introduced and promoted with the eventual hope, according to the pilot report, of being able to do the same worldwide. caBIG also has a collaboration in place with the UK organisation NCRI to exchange technologies and research data. The European Association for Cancer Research, a member association for cancer researchers, conducted an online survey in January 2011 to identify the penetration of the ambitious caBIG project into European laboratories. The survey was sent to 6396 researchers based in Europe, with 764 respondents, a total response rate of 11.94%.

## European survey

A list of caBIG branded tools was given, along with a link to each tool’s web page, and respondents were asked to state how often they used each one: ‘Often’, ‘Sometimes’ or ‘Never’. If they use the tool, respondents were asked to rate it out of 5 (from ‘1 = very poor’ to ‘5 = very good’) and suggest any improvements that could be made.

## Results

### Frequency of use

**Figure 1:**
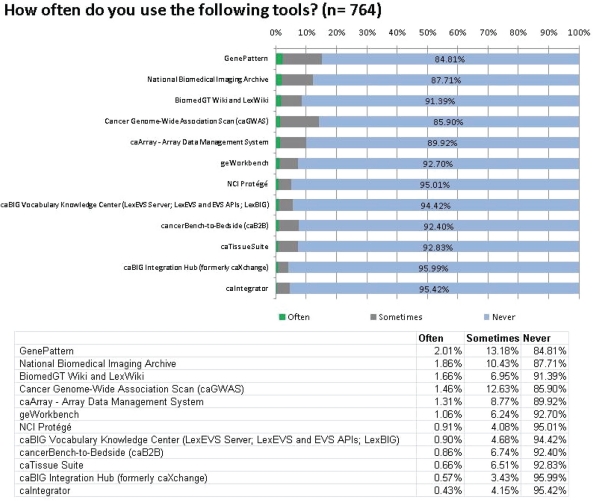
Frequency of use of the caBIG tools.

The tools are listed in descending order in Figure 1 according to the percentage of respondents who answered that they use the tool ‘Often’. It clearly shows that the majority of respondents (ranging between 84.81% and 95.99%) never use the caBIG tools they were asked about. The most frequently used tool, GenePattern, boasts just 2.01% of respondents using it often, while 84.81% never use it. GenePattern is not one of the tools developed exclusively for caBIG: it was developed by the Broad Institute in 2004, accessible from the Broad Institute website, and was made compatible with the caBIG architecture as one of several collaborations: its availability elsewhere and number of collaborations may help to explain its popularity.[3,4] One possible contributing reason for the lower usage of some of the tools could be the fact that many, such as cancerBench-to-Bedside, NCI Protege, caIntegrator, caBIG Integration Hub and the caBIG Vocabulary Knowledge Center, all at the lower end of the usage scale, are designed to be used alongside other caBIG applications, which limit their potential user base to only those who choose to use other tools from caBIG. Because of the intention of the project, to bring together all cancer data in an integrated environment, with some caBIG tools, the outward appearance seems to be ‘all or nothing’—if a user wishes to adopt them, they should do so alongside other caBIG tools, which may be off-putting to some, especially if their lab has already purchased commercial software to do the same tasks. Though all of the caBIG tools are laudably produced in open source code, with an open source license that allows users to adapt them to their own systems and existing software, not all laboratories have the resources or programming knowledge to do this. In November 2010, the NCI Board of Scientific Advisors created an ad hoc working group to provide an independent review of the caBIG program. Their final report, published in March 2011, mentioned that “a significant level of technical knowledge and dedicated local informatics resources are required to make the tools useful in a cancer center’s research environment and to support their customization, adoption and use. This is very frustrating to potential users.”

### Perceived quality of the tools

**Figure 2:**
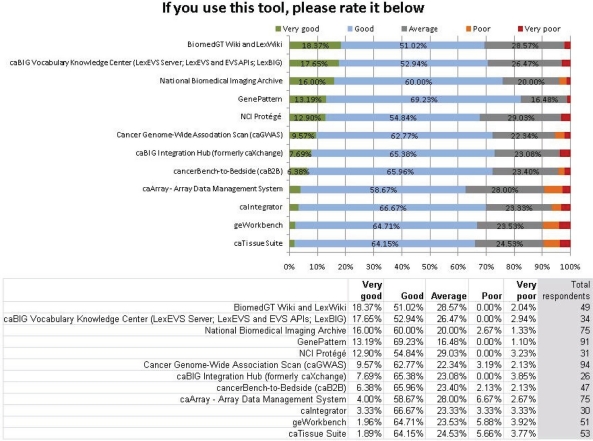
Perceived quality of the caBIG tools.

In Figure 2, though the results show that the tools in question are used very little by the survey respondents, it also shows a high level of regard from those who do use them. Due to the low usage previously demonstrated, the sample sizes for this question were small (see ‘Total respondents’ column on the right). Though there is a clear hierarchy of which tools more highly rated, it is also clear that all of the tools are well regarded by the respondents who use them, with a small minority in each case rating a tool as ‘poor’ or ‘very poor’.

Of course, there is some inherent bias in these responses, as the respondents to this question already use the tools in question. Unless compelled to use them by their institutes or by financial necessity, the very fact that they use a certain tool indicates that it must be of some use to them. The only way to correct this bias would be to conduct a thorough testing of the various tools by researchers who have never used them, and then solicit a rating based on this assessment.

In addition to receiving the most use, GenePattern also received the highest average rating by the respondents. This result was echoed in the March working group 2011 report, where GenePattern received the most positive reports from interviewees.[5]

### Average rating vs. frequency of use

**Figure 3:**
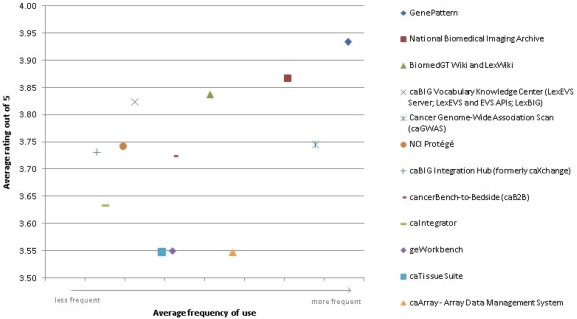
Average rating vs. frequency of use.

In order to better analyse the caBIG tools’ usage and rating, a scatter graph was created to compare these two (Figure 3). The frequency of use responses ‘Often’, ‘Sometimes’ and ‘Never’ were given numerical values in order to reach a weighted average figure for each tool. These numerical values have not been shown on the X axis as it is a meaningless scale: for actual frequency of use figures, see the ‘Frequency of use’ chart. In the above graph, the average frequency of use figure was plotted against the average rating out of 5 (1 = very poor to 5 = very good) for each tool.

In general, the trend is that the higher-rated tools are used more and the lower-rated tools are used less. However, the graph identifies some anomalies: caArray receives the lowest average rating by its users, an average of 3.55 out of 5, and yet is the fourth most frequently used. The fact that caArray is directly accessible from the Web, with no download or sign-in required, may be one reason for its frequent use. Conversely, the caBIG Vocabulary Knowledge Center is one of the highest rated tools and also one of the least used. The niche market for the tool may explain this: its web page describes its purpose as “providing access and support to those individuals and institutions interested in making use of or extending caBIG^®^ tools and other vocabulary tools”[6], thus making it potentially very useful, but only to a select few who are interested in working with or developing caBIG or other vocabulary tools.

### Free text: Improvements

After asking to rate each tool if they used it, a free text box was provided to suggest any improvements that could be made. Few comments were received, which are tabulated below.

**Table:** 

**Please suggest any improvements that could be made**
caArray—Array Data Management System
A good tool
My students use it

geWorkbench
Seems an easy tool for array analysis

caTissue Suite
Useful for overall storage/retrieval of complex biospecimens/case series. Maybe less so for smaller numbers of specimens.
We will use it in the near future

Cancer Genome-Wide Association Scan (caGWAS)
Still learning how to use the program
We have preliminary information, plan to use it more frequently in the near future

National Biomedical Imaging Archive
The integration of images of biomedical research with genomics data is a very potent thus intuitive method
Registration should be a quicker process—we often work on tight deadlines!

caIntegrator
Sometimes, array analyses require a broad computing language knowledge such as R. This tool may help these people not familiar with this computing abilities.

cancerBench-to-Bedside (caB2B)
As integrative tool can be very useful

GenePattern
Will get into it in the near future
It would be great to have a few example input files for every type of plug in
Database is represented with included data, not with real situation

caBIG Integration Hub (formerly caXchange)
Plan to use it this year

NCI Protégé
[No Responses]

caBIG Vocabulary Knowledge Center (LexEVS Server; LexEVS and EVS APIs; LexBIG)
[No Responses]

BiomedGT Wiki and LexWiki
Haven’t used enough to have a good idea of rating

General comments
Comment on this whole survey—most of the tools mentioned, I have never heard of. I might use them if I knew about them and what they do. The BiomedWiki tool I came across from Google.
I should say although I don’t use these tools directly, I have a team of researchers that will find such tools useful.
I was never aware of the availability of these tools until now. Definitely will give them a shot.
I do thank you for valuable information of the mentioned NCI tools which I have never heard about before.

## Conclusions

The survey results show a widespread lack of usage and knowledge of the caBIG research-oriented tools in Europe. Those who chose to comment on the survey overall said that they had never heard of them. With a large number of open access biomedical tools being developed across the world and within Europe by institutes and collaborative partnerships, especially in the burgeoning field of bioinformatics,[7] this is unsurprising. While the NCI’s plan to introduce the caBIG network and tools in the US involved promoting, supporting and subsidising their introduction at NCI-funded institutes, European institutes and scientists received no such promotion, so the array of tools offered under the caBIG umbrella appears to have gone largely unnoticed. This limited uptake of the tools does not appear to be confined to Europe. According to the final report of the NCI Scientific Advisory Board’s working group on caBIG, “the majority of the 32 Bench-to-Bench research tools developed by caBIG^®^ under contracts with commercial or academic investigators have had very limited usage and, as a result, have not generated significant impact in the scientific community.” The working group’s recommendations include a moratorium on all current software development and a 1-year moratorium on all future projects. caBIG has now put out an request for information to have these tools moved into the care and development of the public sector. Whilst this programme has been a Federally mandated project aimed at the domestic market, there is clearly an opportunity for better global dissemination irrespective of who eventually becomes their curator. For Europe, the public policy message is clear. Bioinformatic developments need better dissemination and marketing to the cancer research community and more work needs to be put into establishing ongoing needs assessments from the broad church of cancer research.
